# Photodynamic therapy and tumor-treating fields therapy for newly diagnosed glioblastoma

**DOI:** 10.3389/fonc.2025.1556669

**Published:** 2025-02-28

**Authors:** Shinjiro Fukami, Kenta Nagai, Sho Onodera, Yuki Saito, Jiro Akimoto, Michihiro Kohno

**Affiliations:** ^1^ Department of Neurosurgery, Tokyo Medical University, Tokyo, Japan; ^2^ Department of Neurosurgery, Kohsei Chuo General Hospital, Tokyo, Japan

**Keywords:** glioblastoma, photodynamic therapy, talaporfin sodium, tumor-treating fields therapy, newly diagnosed, prognosis

## Abstract

**Introduction and purpose:**

Various treatment methods, including photodynamic therapy (PDT), are used for glioblastoma (GBM), which is an intractable tumor. Our therapeutic strategy for glioblastoma has been based on resection (if possible), and PDT. On the other hand, after tumor-treating fields therapy (TTF) became available, we have actively recommended it to our patients who are eligible for it. In this report, we describe the clinical characteristics and disease course of glioblastoma patients treated by PDT + TTF at our hospital.

**Methods:**

A total of 14 patients with newly diagnosed glioblastoma, who underwent PDT + TTF from the time of insurance coverage of TTF were analyzed. The median age of the patients was 48 years. There were 10 men and 4 women, with a high prevalence of younger men.

**Results:**

The average duration of TTF was 8.9 (1–19) months, and the main reasons for its discontinuation were recurrence of the tumor and scalp problems. The median progression-free survival of the 14 patients who underwent PDT + TTF was 13.4 months, which tended to be longer than that of the 30 patients who underwent PDT without TTF (11 months). Of the 10 patients who relapsed, 2 had local recurrence and 8 had distant or disseminated recurrence. Two patients with local recurrence underwent repeat resection together with PDT. To date, the prognosis for patient survival of PDT + TTF appears favorable, with 6 patients surviving for more than 2 years.

**Conclusion:**

PDT + TTF treatment for newly diagnosed glioblastoma can be performed without any major adverse events, although there are some problems with the continuation of TTF, such as scalp problems and its high cost. More patients who underwent PDT + TTF relapsed with distant and/or disseminated recurrence than local recurrence, suggesting that this treatment strategy targets local recurrence. Our results demonstrate that combination therapy for newly diagnosed glioblastoma with PDT + TTF may prolong the time to recurrence and improve survival outcomes of patients, although the data in this study are preliminary.

## 1 Introduction

Glioblastoma (GBM) has an unfavorable prognosis, with a mean survival time of patients of less than 2 years. Various treatment methods, including those aiming at local tumor control, such as carmustine wafers (Gliadel^®^, Eisai Inc.), have been developed for this intractable tumor (1). Intraoperative photodynamic therapy (PDT) using talaporfin sodium (TPS, Laserphyrin^®^, Meiji Seika Pharma Co., Ltd.) for GBM has been reported to be effective both in experimental and clinical settings (2, 3). The benefit of PDT has been reported not only for newly diagnosed GBM, but also for recurrent GBM and pediatric GBM patients (4, 5). A phase II study testing the effects of PDT using TPS in patients with malignant brain tumors, including GBM, achieved impressive results, in which the 12-month overall survival (OS), 6-month progression-free survival (PFS), and 6-month local PFS after PDT were 95.5%, 91%, and 91%, respectively (6). Based on these results, PDT using TPS for the treatment of primary intracranial malignant tumors was approved in Japan for health insurance coverage, and this therapy is at present used in more than 30 institutions in Japan. We have also performed PDT since 2014, from the early days of its coverage by insurance in Japan. Our therapeutic strategy for GBM is based mainly on resection of the tumor if possible, and PDT. Tumor-treating fields therapy (TTF; Optune^®^, Novocure Inc.), which is an antimitotic treatment, is also a treatment for the local control of GBM (7, 8). TTF has been shown to prolong patient survival, and is used worldwide (9–11). Therefore, after TTF became available in Japan, we have actively recommended it to our patients who are eligible for it. In this report, we describe the clinical characteristics and disease course of GBM patients treated by PDT + TTF at our hospital.

## 2 Materials and methods

### 2.1 Patients and surgery

Fourteen patients with newly diagnosed GBM who underwent PDT + TTF under TTF insurance indication between 2019 and 2024 were included in this study. TTF is basically recommended for patients with a Karnofsky Performance Status (KPS) of 70 or higher. The median age was 48 years, and the ratio of men to women was 10:4, with a high prevalence of younger men. The mean maximum size of the tumor was about 43.9 mm (25 mm to 63 mm). Two patients had lesions located near the language area, and were operated on under awake craniotomy. Thirteen patients were diagnosed as having GBM, isocitrate dehydrogenase (*IDH)*-wildtype, and 1 patient (GBM, *IDH*-mutant) was diagnosed as having astrocytoma, *IDH*-mutant, grade 4, in accordance with the World Health Organization (WHO) 2021 criteria. O6-methylguanine-DNA methyltransferase (MGMT) methylation status was not be analyzed, as it is not testable at our institution, and cannot be outsourced owing to funding problems. This study was conducted in accordance with the Helsinki Declaration, and was approved by the Ethical Review Board of Tokyo Medical University Hospital (study approval no.: T2020-0278). PDT was performed as previously reported (3, 12). The patients received an intravenous injection of TPS at a dose of 40 mg/m^2^ between 22 and 26 hours prior to tumor resection. After the tumor was resected, a 3-minute PDT (664-nm continuous wave, radiation power density at 150 mW/cm^2^, energy density at 27 J/cm^2^) was conducted on the wall of the resected cavity, while avoiding overlap of the irradiation site. Reflective irradiation using a mirror (PDT mirror^®^, Yufu Itonaga Co., Ltd.) was performed when normal irradiation was not sufficient (13). Irradiation was applied over the entire extraction cavity, and the number of irradiation spots ranged from 3 to 16.

### 2.2 Postoperative treatment

Postoperative therapy involved the combined use of temozolomide (TMZ) and radiotherapy (RT) for all patients. TMZ and RZ were started 10 to 20 days after the first operation, when the stitches were removed. The initial therapeutic dose of TMZ was 75 mg/m^2^ for 42 days. RT was performed with extended localized irradiation (60 gray for 30 fractions). Bevacizumab (BEV) treatment was started soon after surgery in patients who had a residual lesion after the initial treatment, and in whom re-resection was considered difficult. BEV was administered intravenously at a dose of 10 mg/kg at 2-week intervals. After the initial treatment, maintenance therapy was performed with TMZ only or with a combination of TMZ and BEV. Maintenance therapy with TMZ was 150 to 200 mg/m^2^ for 5 days per 28 days, and continued until the patient became unable to take it internally or for 12 cycles. Treatment for recurrence is re-resection with PDT as much as possible, if a tumor recurs locally. If the recurrence is at a distant site or is disseminated, BEV treatment is performed in patients not receiving BEV. TTF is recommended for all patients with newly diagnosed GBM who have a KPS of 70 or higher after the initial treatment, and is performed only in patients who provide their consent. All 14 patients also had their family’s cooperation.

### 2.3 Patients not treated by TTF

To investigate the effect of TTF on the prognosis of patients with newly diagnosed GBM, 30 patients with GBM who did not undergo TTF, but underwent PDT and had a KPS of higher than 70 after the initial treatment at our institution between 2014 and 2023 were also analyzed. The reasons for not performing TTF included the lack of insurance coverage, the patient having no wish to undergo TTF, and the judgment of the physician in charge. These patients underwent TMZ, RT, and BEV as adjuvant therapies. The median patient age and the mean maximum size of the tumor were 69 years and 49.6 mm, respectively.

### 2.4 Statistical analyses

Statistical analyses were performed using GraphPad Prism 5 software® (GraphPad Software, Inc.) for the comparison of age, tumor size, KPS, median progression-free survival (mPFS), and median overall survival (mOS). Differences between 2 groups, such as regarding age, tumor size, and KPS were analyzed using the Mann-Whitney test. Differences in survival time were analyzed using the log-rank test. A *p*-value of less than 0.05 was considered to indicate a statistically significant difference between groups.

## 3 Results

The characteristics and clinical course of all the patients are shown in **Table 1**. Two patients had lesions near the speech area, which were excised by awake craniotomy. The extent of tumor removal was gross total removal (95%–99%) in 9 patients, subtotal removal (80%–94%) in 4 patients, and partial removal (20%–79%) in 1 patient. The definitions of the degree of tumor removal have been reported previously (14). Five patients had residual lesions after the initial treatment. One patient underwent re-resection and PDT, whereas 4 patients could not undergo re-resection and were started on BEV biweekly maintenance therapy. All patients had a KPS of more than 70 at the start of the TTF, whereas 13 patients had a KPS of more than 80. The target using time per day and duration of TTF wearing was at least 75% per day for at least 1 month. The actual average duration was 8.9 (1–19) months, and the average daily rate of TTF use was 66% (35%–94%). At the time of writing, 3 patients (no. 9, 12, and 14) are continuing the use of TTF. The reasons for the discontinuation of TTF in the other patients were tumor recurrence with decreased activities of daily living in 5 patients, decreased motivation of TTF wearing in 3 patients, and scalp problems, such as wound rash and infection in 3 patients. No adverse events owing to the use of PDT were observed. A total of 10 out of the 14 patients showed recurrence during the treatment course. Dissemination and/or distant recurrence occurred in 8 of the 10 patients (no. 1, 3, 4, 5, 6, 11,13, and 14), despite favorable local tumor control. Only 2 patients (no. 7 and 10) developed local recurrence and underwent reoperation together with PDT. Both patients survived for more than 2 years (patient 7: 29.5 months; patient 10: 27.9 months) after the initial surgery. Of the 11 patients (no. 1–11) who were followed for more than 2 years from the initial treatment, over half (6/11) have survived for more than 2 years.

**Table 1:** Clinical features of the 14 patients treated by photodynamic therapy (PDT) + tumor-treating fields therapy (TTF).

Patient	Age (year)/sex	Tumor location	Pathology	Size (mm)	Times of PDT	Removal method	Adjuvant treatment	KPS	TTF duration (months)	TTF mean time (%/day)	PFS (months)	OS (months)	Recurrence pattern	Patient status at time of writing
1	40-45/M	Rt Parietal	GBM *IDH*-wild	46	×16	Gross total (95%–99%)	RT+TMZ	70	4	72	7.1	10	Dis.	Dead
2	50-54/M	Lt Temporal	GBM *IDH*-wild	30	×8	Gross total (95%–99%)	RT+TMZ	80	17	65	54.4	54.4	(–)	Alive
3	30-35/M	Lt Occipital	GBM *IDH*-mutant	33	×10	Subtotal (80%–94%)	RT+TMZ	80	7	55	7.8	10.9	Dis.	Dead
4	50-55/M	Rt Frontal	GBM *IDH*-wild	63	×14	Gross total (95%–99%)	RT+TMZ+BEV	80	4	71	7.3	10.2	Dis.	Dead
5	40-45/F	Lt Frontal	GBM *IDH*-wild	51	×8	Subtotal (80%–94%)	RT+TMZ+BEV	80	12	75	9.8	18.1	Dis.	Dead
6	45-50/M	Lt Frontal	GBM *IDH*-wild	27	×4	Partial (20%–79%)	RT+TMZ+BEV	80	12	72	14.9	17.5	Dis.	Dead
7	45-50/M	Rt Temporal	GBM *IDH*-wild	61	×5	Gross total (95%–99%)	RT+TMZ	80	18	86	11.8	29.5	Local	Alive
8	60-65/M	Lt Frontal	GBM *IDH*-wild	41	×4	Gross total (95%–99%)	RT+TMZ	90	4	35	(–)	28.3	(–)	Alive
9	40-45/F	Lt Temporal	GBM *IDH*-wild	51	×5	Subtotal (80%–94%)	RT+TMZ+BEV	80	19	43	(–)	29.1	(–)	Alive
10	55-60/F	Lt Frontal	GBM *IDH*-wild	28	×3	Gross total (95%–99%)	RT+TMZ	80	1	94	4.2	27.9	Local	Alive
11	30-32/M	Lt Frontal	GBM *IDH*-wild	51	×5	Gross total (95%–99%)	RT+TMZ	90	7	58	20	24	Dis.	Alive
12	65-70/F	Rt Temporal	GBM *IDH*-wild	54	×7	Subtotal (80%–94%)	RT+TMZ	90	7	79	(–)	17.3	(–)	Alive
13	70-75/M	Rt Temporal	GBM *IDH*-wild	25	×5	Gross total (95%–99%)	RT+TMZ	90	8	61	15.8	16.8	Dis.	Alive
14	35-40/M	Rt Parietal	GBM *IDH*-wild	54	×5	Gross total (95%–99%)	RT+TMZ	90	4	59	9.4	10.3	Dis.	Alive

Rt, right; Lt, center; M, male; F, female; GBM, glioblastoma; *IDH*, isocitrate dehydrogenase; RT, radiation therapy; TMZ, temozolomide; BEV, bevacizumab; KPS, Karnofsky Performance Status; PFS, progression-free survival; OS, overall survival; Dis., dissemination or distant recurrence.

**Figure 1:**
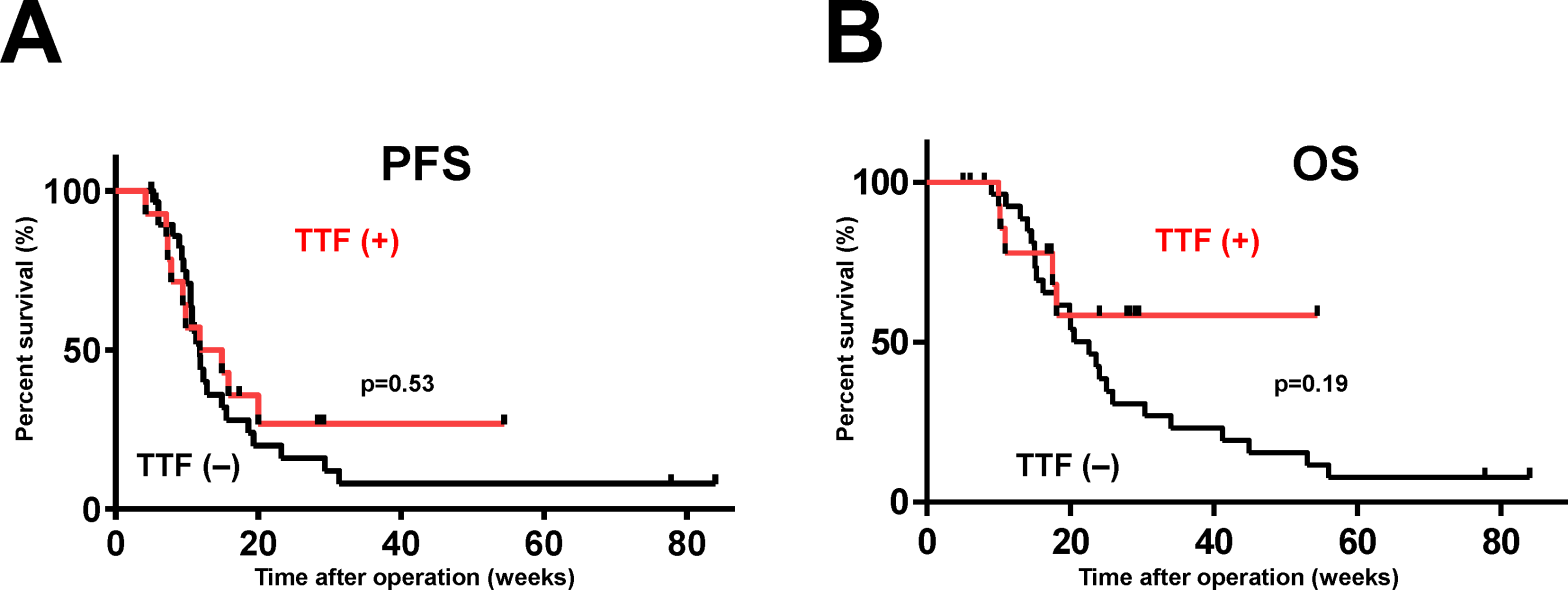
Survival curves of patients with newly diagnosed GBM treated by PDT with or without TTF. The vertical axis of the graph is the rate of survival of the patients (%) and the horizontal axis is the time since the initial operation (weeks). mPFS **(A)** and mOS **(B)** were comparatively more favorable in patients who underwent TTF, although the differences were not statistically significant (mPFS, *p* = 0.53; mOS, *p* = 0.19).

The prognosis was compared between the 14 patients treated by PDT + TTF and the 30 patients treated by PDT + no TTF (**Table 2**). The median age was 48-years old for patients treated by PDT + TTF, and 69-years old for patients treated by PDT + no TTF, indicating that patients treated by TTF were younger (*p* = 0.003), but there were no statistically significant differences in mean KPS (83 [PDT + TTF] vs. 78 [PDT + no TTF]) and maximum tumor diameter (43.9 cm [PDT + TTF] vs. 49.6 cm [PDT + no TTF]) at the time of the start of TTF. Gross total resection was achieved in 64% (9/14) of patients who underwent TTF, which was lower than the 87% (26/30) of patients who did not undergo TTF. The mPFS for patients treated by TTF was 13.4 months, which tended to be longer than that for patients not treated by TTF (11.8 months), although the difference was not statistically significant (hazard ratio = 1.225, 95% confidence interval: 0.6123–2.573, *p* = 0.53, **Figure 1A**). Although it is difficult to evaluate the OS of patients who underwent TTF because they did not reach the median, there was a tendency for more patients who underwent TTF to have prolonged survival from 20 months after surgery than those who did not undergo TTF (hazard ratio = 1.732, 95% confidence interval: 0.7569–3.96, *p* = 0.19, **Figure 1B**).

**Table 2:** Comparison of 44 glioblastoma (GBM) patients treated by PDT together with or without TTF.

	TTF (+) (n = 14)	TTF (−) (n = 30)	*p*-value
Operation period (AD)	2019–2024	2014–2023	
Median age (years)	48	69	0.003* Mann-Whitney test
Mean maximum tumor size (mm)	43.9	49.6	0.165 Mann-Whitney test
Pathology			
GBM, *IDH*-wild	13	26	
GBM, *IDH*-mutant	1	3	
GBM, NOS	0	1	
Resection			
Partial (20%–79%)	1	1	
Subtotal (80%–94%)	4	3	
Gross total (95%–99%)	9	26	
Mean KPS at the start of TTF	83	78	0.063 Mann-Whitney test
mPFS (months)	13.4	11.8	0.53 Log-rank test
mOS (months)	Not reached	22.6	0.19 Log-rank test

AD, *Anno Domini*; TTF, tumor-treating fields therapy; PDT, photodynamic therapy; GBM, glioblastoma; NOS, not otherwise specified; *IDH*, isocitrate dehydrogenase; KPS, Karnofsky Performance Status; mPFS, median progression-free survival; mOS median overall survival; *p < 0.05.

### 3.1 Two representative cases of patients treated by PDT + TTF

#### 3.1.1 Case 9

**Figure 2:**
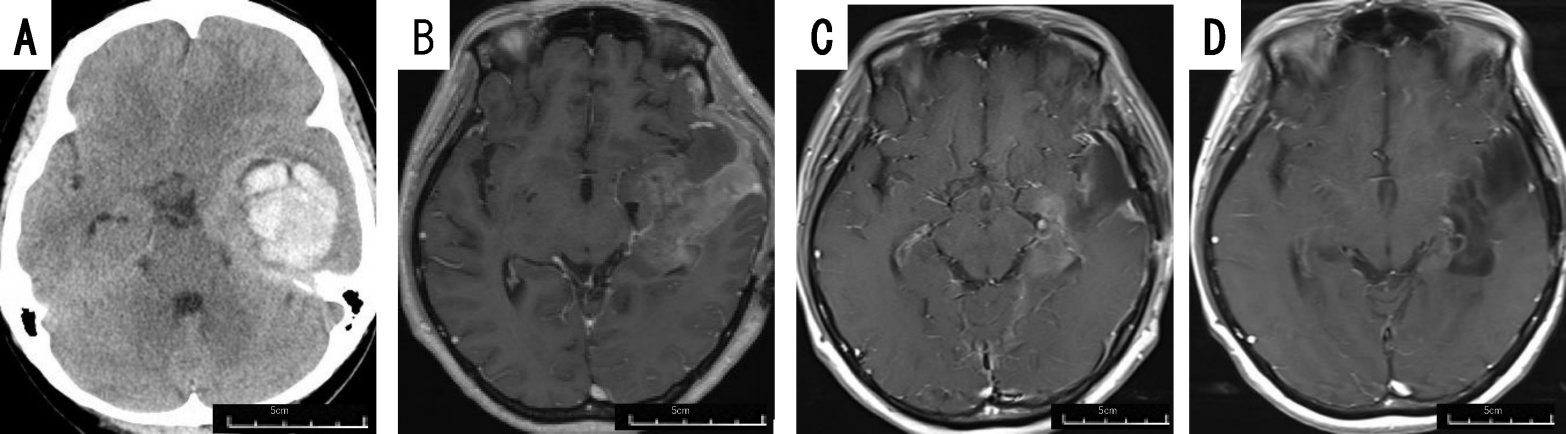
Patient 9 (40-45 year-old woman): GBM, *IDH*-wild, presenting with intratumoral hemorrhage. Preoperative computed tomography image **(A)** of patient 9 displaying intracerebral hematoma in the left temporal lobe, requiring evacuation. Gadolinium-enhanced brain MRI taken immediately before tumor removal **(B)**, and 1 month **(C)** and 24 months **(D)** after tumor removal.

The patient was a 40-45 year-old woman. She developed impaired consciousness, and was found to have an intracerebral hematoma in the left temporal lobe (**Figure 2A**). She initially underwent surgery for evacuation of the hematoma, and a tumor was detected in the hematoma intraoperatively. The tumor was diagnosed as GBM *IDH*-wild, and she was referred to our hospital. She had no motor weakness, but slight motor aphasia. The tumor was located mainly in the medial part of the left temporal lobe (**Figure 2B**). Therefore, we performed tumor removal with PDT under general anesthesia, rather than awake craniotomy, because she developed slight motor aphasia. Because the tumor was invading the internal side of the left temporal lobe including the hippocampus, we could not achieve total tumor removal. After the operation, she received chemoradiotherapy with TMZ and RT as the initial postoperative treatment. One month after the operation, the residual tumor remained in the medial temporal lobe (**Figure 2C**). Therefore, biweekly BEV was started together with TMZ therapy. One year after the operation, combination therapy with TMZ and BEV were terminated, but the tumor has not recurred 24 months after surgery (**Figure 2D**), upon the continuation of TTF.

#### 3.1.2 Case 11

**Figure 3:**
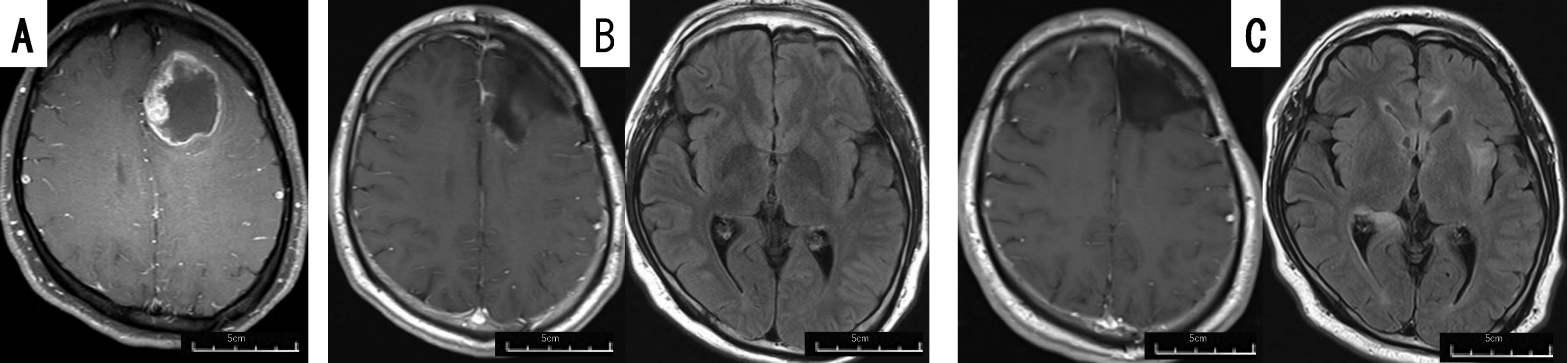
Patient 11 (30-35 year-old man): GBM, *IDH*-wild, demonstrating dissemination 20 months after PDT+TTF. Preoperative gadolinium-enhanced brain MRI **(A)** of patient 11, displaying a lesion in the left frontal lobe. Postoperative gadolinium-enhanced (left images) and FLAIR (right images) brain MRI taken 2 months **(B)** and 20 months **(C)** after the operation. The brain FLAIR MRI taken 20 months after the operation displayed dissemination around the lateral ventricle and left insular lobe.

The patient was a 30-35 year-old man. He presented with a headache and personality changes. No motor paralysis was observed. Initial magnetic resonance imaging (MRI) displayed an irregular cystic enhanced tumor in the left frontal lobe (**Figure 3A**). Gross total removal and PDT was performed, which resulted in the disappearance of personality changes. MRI after treatment with TMZ + RT displayed no residual tumor, with no signal change in the periventricular region on fluid attenuated inversion recovery (FLAIR) (**Figure 3B**), and maintenance therapy with TMZ and TTF, but no BEV, was started. The patient was able to work postoperatively, but 20 months after the initial operation, he developed tumor dissemination in the periventricular region, without local recurrence (**Figure 3C**), and began treatment with BEV. He is alive 24 months after the initial operation.

## 4 Discussion

Our present results showed that mPFS was prolonged by about 2 months in patients who underwent TTF, and OS also tended to be prolonged in patients who survived for longer than 20 months. Although there were no statistically significant differences owing to the small number of patients and the short follow-up period, and our results are preliminary, a moderately positive effect of TTF was also observed in the patients who underwent PDT. The effect of the approximately 2-month extension of mPFS was similar to the results of a prospective study on the effects of TTF (by 2.7 months) (9). Regarding the effect of PDT, mOS increased significantly (by about 5 months) in the series by Nitta et al., in which total resection was possible in the patients, and in our patient series, in which total resection was not possible in all patients, both mPFS and mOS increased, although the differences were not statistically significant (12, 15). In addition, Fujimoto et al. performed PDT in 44 patients, and their OS increased by more than 7 months compared with the controls (16). Although it is not a simple calculation, we would expect PDT + TTF to prolong mPFS and mOS by 6 to 12 months, respectively. Regarding mPFS, in the EF-14 trial, which is a prospective study of TTF, patients treated by TMZ only had a mPFS of 7.8 months from diagnosis (9). The mPFS in the present study was 13.4 months, and the extension by PDT + TTF was about 6 months, which was considered to be as expected. The data from the patient series of Nitta et al. in which total resection was possible, and our patient series in which total resection was not possible suggest that PDT prolongs PFS and OS early in the course of treatment, by up to 10 to 20 months after the start of treatment (12, 15). In the data of the present study, there is almost no difference in PFS and OS between patients treated by TTF and those not treated by TTF up to 20 months after treatment, suggesting that TTF may be effective in prolonging survival beyond that time. These data suggest that PDT delays early recurrence, and TTF may delay subsequent recurrence. This data is consistent with the relatively long-term survival of 50% of patients who could be followed for more than 2 years. The mechanism of the interaction of PDT and TTF is unclear. Regarding their direct association, the mechanism of the effect of PDT is thought to be local control in the acute postoperative period, from 1 day to several weeks postoperatively, whereas the mechanism of the effect of TTF is chronic local control in a maintenance therapy-oriented manner. Although one-third of the patients in our present study could not undergo total resection and had to undergo subtotal or partial resection, the prognoses of these 5 patients did not appear to be unfavorable (died after 10.9 months, died after 18.1 months, died after 17.5 months, alive after 29.1 months, and alive after 17.3 months). The EF-14 trial showed that the median OS of patients was prolonged with the use of TTF, regardless of the tumor removal status, and even with biopsy or partial removal (9). The data from our institution demonstrating that PDT is also effective for GBM that is difficult to remove entirely, and can only be removed subtotally or partially, suggests that PDT + TTF is more useful for patients in whom total resection is difficult, such as those with tumors in eloquent areas or with large tumors (12).

Our analysis of recurrence patterns in this study showed that local recurrence after PDT + TTF was uncommon, and there was a tendency towards distant and disseminated recurrence (8/10). Several research groups in Japan, including ours have reported more distant and/or disseminated recurrences after tumor removal with PDT than without PDT for newly diagnosed GBM (12, 15, 16). The recurrence pattern was thought to be dominated by dissemination and/or distant recurrence because both TTF and PDT target local recurrence. The laser used in TPS is thought to reach a depth of about 4 to 5 mm in the normal brain, and a depth of about 10 mm in edematous brain structures in regions of tumor invasion (17). Our analysis of corpse specimens indicated that the antitumor effect of PDT is expected to extend to a depth of 8 to 10 mm. The histopathological changes of tissues in the PDT area occur in regions of 9- to 18-mm in depth (18). Therefore, it is essential to reduce dissemination and/or distant recurrence to expect a more favorable prognosis. For this purpose, it is necessary to develop novel systemic therapies, such as molecular-targeted drugs and new immunotherapies, in addition to surgical innovations, such as not opening the ventricles or removing them *en bloc* as much as possible. There are reports demonstrating the efficacy of TTF and immunotherapy (19), and we hope that the addition of PDT to these therapies will effectively suppress local recurrence and dissemination/distant recurrence, and extend the survival time of patients. Recently, the usefulness of interstitial PDT (iPDT), which is a procedure using 5-aminolevulinic acid under fiber optics, has been reported as a minimally invasive method of PDT for the treatment of unresectable GBM (20–22). We have also reported the usefulness of iPDT using TPS *in vivo* in rodents (23, 24). Therefore, iPDT using an optical fiber, and TTF treatments that do not require craniotomy have the potential to be developed as minimally invasive therapies in the future.

The prognosis of GBM is expected to improve gradually in the future, by making full use of multiple modalities, including PDT and TTF, for its treatment. However, the cost of each modality is expensive, and this can be problematic from a healthcare economic point of view. Cost reductions of PDT and TTF are unlikely to be achieved in Japan because GBM is a rare cancer, accounting for 1.68 out of 100,000 people per year (25). In the future, further research and indications for other organs, such as lung and esophageal cancer for which PDT is covered by insurance in Japan, may reduce the cost of the use of PDT + TTF owing to the economies of scale. We hope that more research on the use of TTF for other organs besides brain tumors will be performed in the future (26, 27). TTF is an electric field therapy that suppresses cell division, and no alternative treatment is currently available. A major problem of TTF for brain tumors is the need to shave the scalp every day and scalp problems, such as wound rash and infections, caused by the adhesive. In addition to these problems, there is also a psychological burden in shaving the scalp, particularly for women. New devices that do not require shaving, such as hats and headgear, are desirable in the future.

The limitations of this study are the small number of patients and the short observation period, making the results insufficient for statistical analysis. In the future, if the use of PDT with TPS spreads worldwide, the effectiveness of its combination with TTF, which is already used in many institutions, will become clear. The present study addresses the demonstrates that PDT + TTF can be performed safely and shows a favorable tendency.

## 5 Conclusion

In this study, the effect of PDT + TTF on newly diagnosed GBM was investigated. This strategy can be performed without any major adverse events, although there are some problems with the continuation of TTF, such as scalp problems and its high cost. More patients relapsed with distant and/or disseminated recurrence than local recurrence, suggesting that this treatment strategy targets local recurrence. Our results demonstrate that combination therapy for newly diagnosed GBM with PDT and TTF may prolong the time to recurrence and improve survival outcomes, although the data in this study are preliminary. Further studies with a larger number of patients matched for age, degree of resection, and MGMT methylation status are needed to validate our results.

## Data Availability

The original contributions presented in the study are included in the article/supplementary material. Further inquiries can be directed to the corresponding author.
